# SSR Linkage Maps and Identification of QTL Controlling Morpho-Phenological Traits in Two Iranian Wheat RIL Populations

**DOI:** 10.3390/biotech11030032

**Published:** 2022-08-08

**Authors:** Hossein Sabouri, Sharifeh Mohammad Alegh, Narges Sahranavard, Somayyeh Sanchouli

**Affiliations:** 1 Department of Plant Production, College of Agricultural Science and Natural Resources, Gonbad Kavous University, Gonbad Kavous 4971799151, Iran; 2 Department of Biology, College of Science, Gonbad Kavous University, Gonbad Kavous 4971799151, Iran

**Keywords:** wheat, QTL, mapping, marker-assisted selection

## Abstract

Wheat is one of the essential grains grown in large areas. Identifying the genetic structure of agronomic and morphological traits of wheat can help to discover the genetic mechanisms of grain yield. In order to map the morpho-phenological traits, an experiment was conducted in the two cropping years of 2020 and 2021 on the university farm of the Faculty of Agriculture, GonbadKavous University. This study used two F8 populations, including 120 lines resulting from Gonbad × Zagros and Gonbad × Kuhdasht. The number of days to physiological maturity, number of days to flowering, number of germinated grains, number of tillers, number of tillers per plant, grain filling periods, plant height, peduncle length, spike length, awn length, spike weight, peduncle diameter, flag leaf length and weight, number of spikelets per spike, number of grains per spike, grain length, grain width, 1000-grain weight, biomass, grain yield, harvest index, straw-weight, and number of fertile spikelets per spike were measured. A total of 21 and 13 QTLs were identified for 11 and 13 traits in 2020 and 2021, respectively. In 2020, qGL-3D and qHI-1A were identified for grain length and harvest index on chromosomes 3D and 1A, explaining over 20% phenotypic variation, respectively. qNT-5B, qNTS-2D, and qSL-1D were identified on chromosomes 5B, 2D, and 1D with the LOD scores of 4.5, 4.13, and 3.89 in 2021, respectively.

## 1. Introduction

Wheat (*Triticumaesivum* L.) is a critical crop cultivated in a wide range of areas and is one of the main sources of carbohydrates, protein, fiber, amino acids, minerals, and vitamins. Wheat provides about 20% of the total protein and calories needed daily by 4.5 billion people worldwide [1,2,3]. Reducing agricultural farms and climate change is a significant challenge in supplying wheat to the world’s growing population [4]. Therefore, there is an urgent need to increase wheat productivity, and growing high-yielding wheat cultivars is one of the main strategies to increase total food production [5]. The main traits that determine the yield of wheat are the number of spikes, the number of grains per spike, and the weight of 1000 grains. In addition, spike length and the number of spikelets per spike, as well as the length, width, and area of flag leaf, greatly affect yield [6,7,8,9]. 

Significant advances in molecular biology and biostatistics have led to identifying several genes for grain-yield-related traits [10,11,12,13].

Identifying the genetic structure of yield-related traits classified as quantitative traits can help to discover the genetic mechanisms of grain yield [4,14,15,16].

Using different molecular markers and linkage maps in wheat, it is possible to identify markers associated with traits and use them in marker selection programs [14,17,18,19,20].

Hu et al. [4] have identified 161 quantitative trait loci (QTLs) for yield-related traits, including grain yield per plant; the number of spikes per plant; the number of kernel per spike; spike length; the number of spikelet per spike; flag leaf length; flag leaf width; flag leaf area; plant height; anthesis date; and date heading on 21 chromosomes except for 2D, 3D, and 6D. Chopra et al. [21] have evaluated a population of 206 recombinant inbred lines (RILs) leading to wheat cultivars WL711 and C306 under drought stress conditions (F8 and F10). Major QTLs, including qFLAWD.2D.1, qCMSWD.3B.3, and qCMSWD.3B.3, for flag leaf area, flag leaf length, flag leaf width, and cell membrane constant were identified on chromosomes 2DS and 3BS. Liu et al. [22] have examined a population of 276 RILs resulting from SYN-D × Weebill1 under drought and heat stresses. Finally, 71 QTLs were identified for the study traits. Five QTLs for yield and traits related to drought, heat, and drought tolerance were identified on chromosomes 2A, 3D, 6D (two QTLs), and 7B.

Owing to the importance of identifying QTLs controlling yield and yield-related traits, the present study was conducted to detect genes controlling quantitative traits in RIL populations of Iranian wheat resulting from Zagros × Gonbad and Kuhdash × Gonbad in 2020 and 2021.

## 2. Materials and Methods

This study was carried out in 2020 and 2021 in the research farm of Gonbad Kavous University at Latitude and Longitude 37°15′0″ north and 55°10′2″ east, with an altitude of 46 m above sea level, which has a warm and semi-arid Mediterranean climate based on the climatic classification of Koppen.

**Figure 1:**
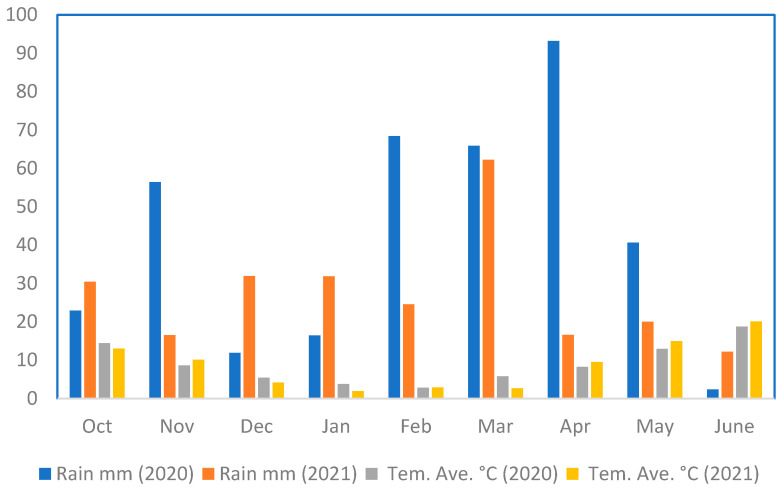
Rainfall and temperature in the experimental region.

The rain and temperature values in 2020 and 2021 are presented in Figure 1, and other meteorological statistics are given in Tables S1 and S2. In this study, 120 lines of two F8 RIL populations resulting from Zagros × Gonbad and Kuhdasht × Gonbad were cultivated according to the alpha lattice design. Planting rows were 2 m in length, and the row spacing was 20 cm. The number of germinated seeds (NGS), the number of days to flowering (NDF), the number of days to physiological maturity (NDM), grain filling period (GFP), the number of tillers (NT), plant height (PH), peduncle length (PDL), peduncle diameter (PD), spike length (SPL), spike weight (WSP), flag leaf length (FLL), flag leaf width (FLW), the number of spikelets per spike (NSSP), the number of fertile spikelets per spike (NFSP) the number of grains per spike (NGSP), grain length (GL), grain width (GW), 1000-grain weight (TGW), awn length (AWL), biomass yield (BYI), grain yield (GYI), straw-weight (STW), and harvest index (HI) were measured.

In order to extract DNA, young leaves of 120 lines from each population along with the parents were used, and then genomic DNA was extracted according to the modified cetyltrimethylammonium bromide (CTAB) method [23]. A polymerase chain reaction was performed for 600 simple sequence repeat (SSR) primers (https://wheat.pw.usda.gov/GG3/, accessed on 30 March 2020) for each population using a 10 μL BioRad thermocycler. PCR solution contained 1X PCR buffer, 0.25 μL MgCl_2_, 1 μldNTPs, 0.5 μL of each primer with 5 mM concentration, Taq polymerase, and 50 ng template DNA. After 5 min of denaturing at 94 °C, 35 cycles were performed, including 1 min at 94 °C, 45 s at an annealing temperature of 55 °C, 1 min at 72 °C, and final expansion for 7 min at 72 °C. The amplification products were separated by electrophoresis in a 3% agarose geland visualized under UV after staining with ethidium bromide or electrophoresis in a 6% polyacrylamide gel visualized by a simplified silver staining method [24].

A total of 689 SSR primers were used in this research. These SSR primer pairs were surveyed based on their polymorphism between two parents, and the primers exhibiting polymorphism were used to amplify the DNA of each plant of the RIL population.

All polymorphic SSR markers were evaluated with the χ^2^ test against a 1:1 segregation ratio at a 0.01 probability level using the QGene program [25]. Linkage analysis was conducted with Map Manager QTX17 [26] for the segregating polymorphic markers. The maximum-likelihood map order for the markers was determined with a logarithm of the odds (LOD) score threshold of 3.0, and used as a fixed sequence framework for integrating the linkage data from the population. All map distances (centi Morgan) were reported in Kosambi units [27], and the critical LOD score thresholds of 3.0 and 0.05 were used to determine the linkage groups and calculate map distances. Lander and Botstein [28] have established an interval mapping framework for mapping QTLs. The genome-wide composite interval mapping (gCIM) was applied to identify QTLs and examine their effects, and the point with the highest LOD was identified as the area with the highest probability of QTL. Chromosome walking was performed at 2 cm, and a LOD score of 2.5 was considered as a threshold. The QTL.gCIMapping.GUI v2.0 package was used for gCIMapping methods with R software [29].

## 3. Results

### 3.1. Gonbad Zagros RIL Population

#### 3.1.1. Phenotypic Evaluations

The frequency distribution of phenotypic values of the study traits is shown in Figures S1 and S2 for the population resulting from Gonbad × Zagros in 2020 and 2021, respectively. The phenotypic distribution of traits was continuous and normal, a reason for the quantitative inheritance of the study traits. 

**Figure 2:**
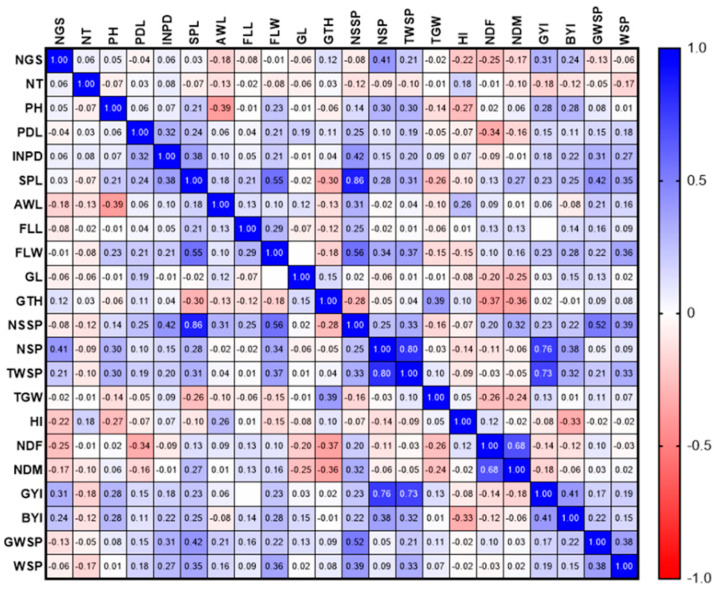
Correlation of study traits in F8 wheat lines resulting from Gonbad × Zagros in 2020.

**Figure 3:**
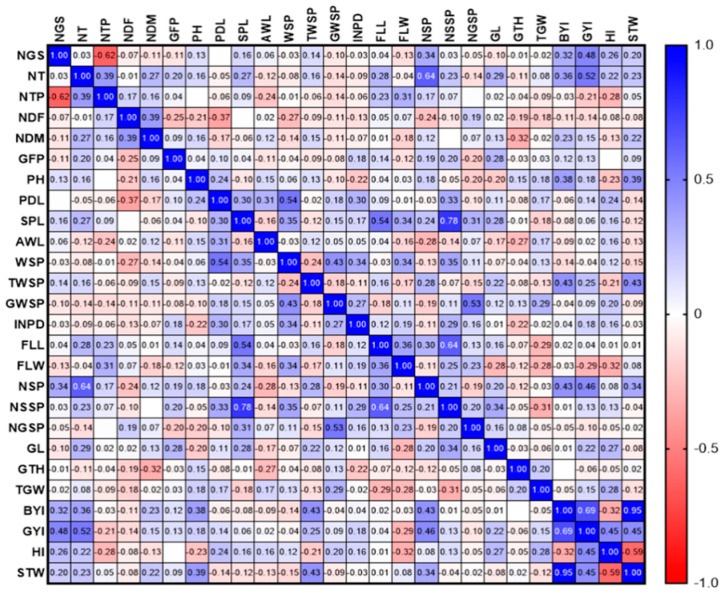
Correlation of study traits in F8 wheat lines resulting from Gonbad × Zagros in 2021.

The GYI had a direct and significant relationship with NT, BYI, and the TWSP in 2020 and 2021. Moreover, in 2020, a positive and significant relationship was observed between FLW, NSSP (0.556 **), NGSP (0.864 **), and GWSP (0.518), as well as SPL and FLW (0.53 **) (Figure 2). In 2020, a positive and significant correlation was observed between NSSP, SPL, and FLL. There was also a positive relationship between WSP and PDL (Figure 3).

The GYI in both years was directly and significantly correlated with NT, BYI, and WSP. Stepwise regression was used to select the traits that critically affect grain yield. In 2020, NSP and TGW explained the most changes in GYI (Table 1). However, in 2021, NT, NGS, STW, FLW, and INDP formed the regression model and explained 69.50% of phenotypic variation in the GYI (Table 2).

**Figure 4:**
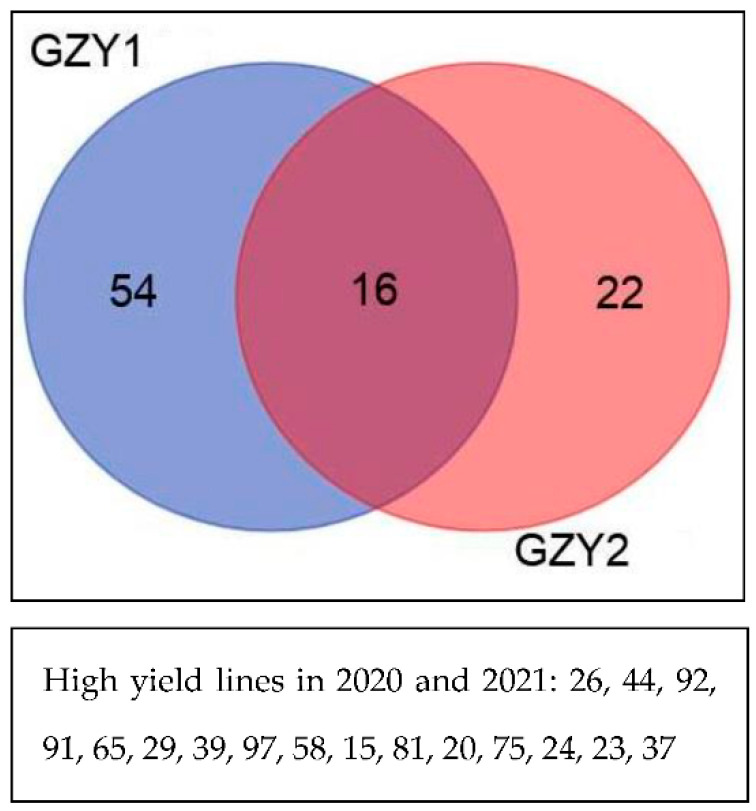
Classification of F8 wheat lines resulting from Gonbad × Zagros in 2020 and 2021.

To group the study lines, cluster analysis was performed based on the grain yield. In both years, the lines were divided into two groups. Lines 26, 44, 92, 91, 65, 29, 39, 97, 58, 15, 81, 20, 75, 24, 23, and 37 had high performance in both years, while line 54 and line 22 had higher performance in 2020 and in 2021, respectively (Figure 4).

Examining the reaction of lines caused by Gonbad × Zagros crosses by considering significant traits in 2020 using Biplot analysis showed that lines 31, 30, 30, 20, and 26 were the most valuable in terms of PH, NDF, TGW, GYI, and NSP. Considering the traits of PH, NDF, TGW, GYI, and NSP, genotypes 10 and 66 were selected as the best cultivars. NDF and TGW show the most diversity for the examined lines.

**Figure 5:**
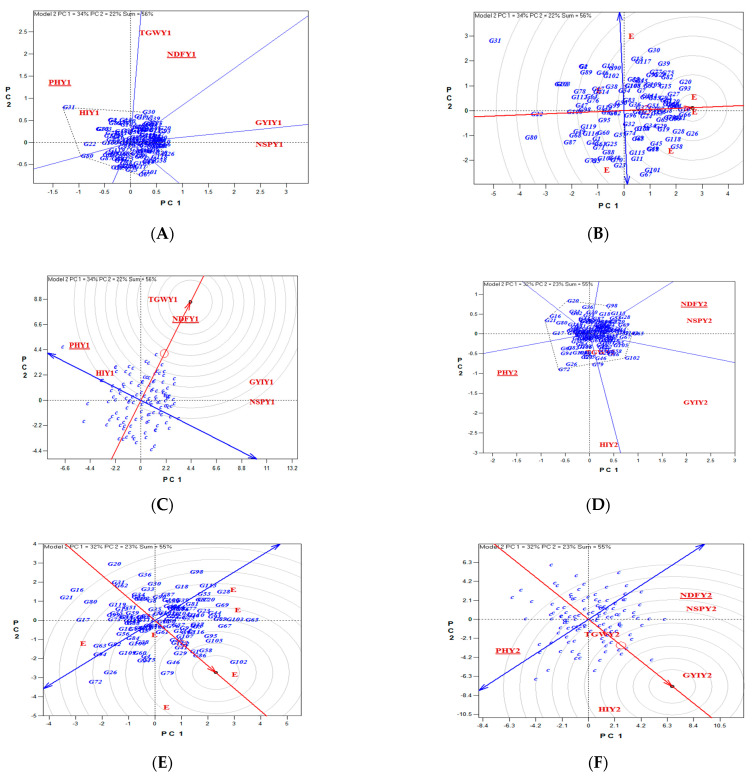
The reaction of lines caused Gonbad × Zagros crosses by considering significant traits in 2020 and 2022 using Biplot. Identifying genotypes with higher values for traits (**A**,**D**), determining the best genotype considering significant traits (**B**,**E**), and identifying the most important traits affecting genetic diversity (**C**,**F**).

Moreover, Biplot analysis in 2021 showed that lines 72, 65, 79, 102, and 65 were better in terms of PH, NDF, TGW, GYI, and NSP. Considering these traits, genotype 102 was selected as the best cultivar. The traits of GYI and HI were the most diverse for the studied lines (Figure 5).

#### 3.1.2. Genotypic Evaluations

**Figure 6:**
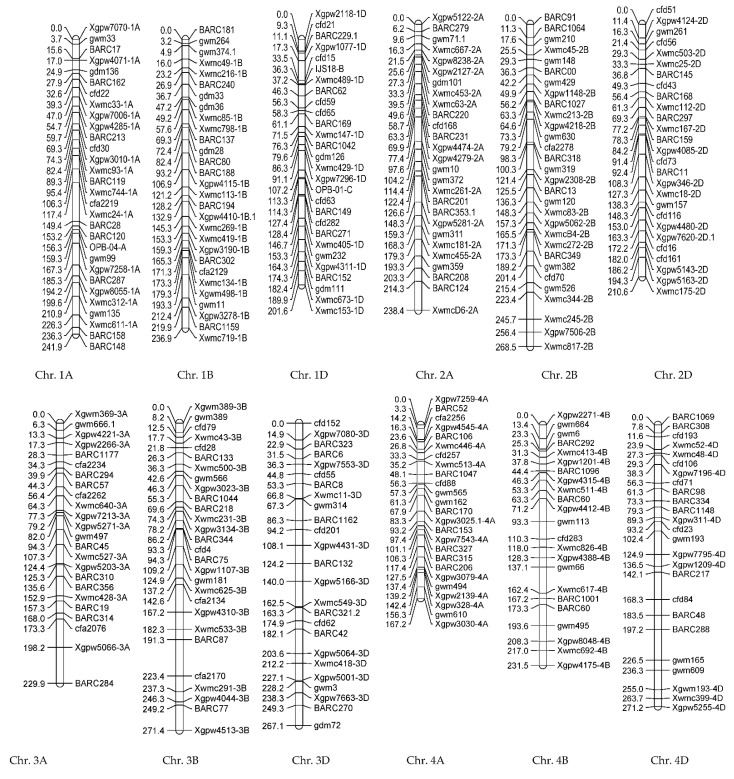

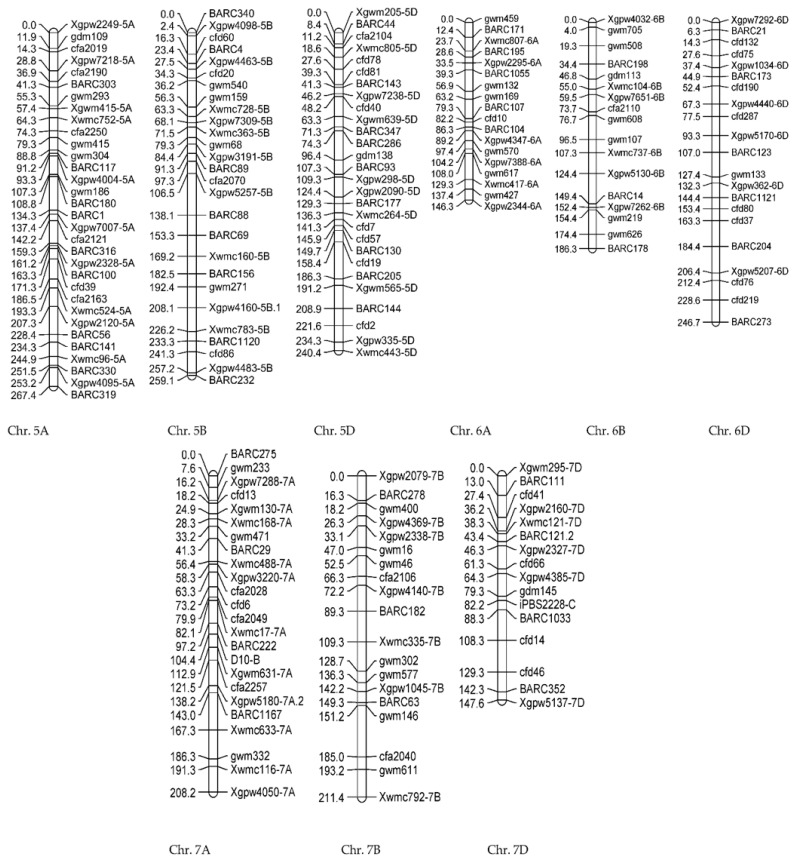
SSR linkage map developed using F8 wheat lines resulting from Gonbad × Zagros.

The linkage map in the population derived from Gonbad × Zagros was created using 523 SSR markers on 21 wheat chromosomes. This map covered 4749.6 cm of the wheat genome. The marker distances for genomes A, B, and D were 6.1, 6, and 6.2 cm, respectively. The length of genome A was 1499.3 cm, and those of genomes B and D were 1665.1 and 1585.2 cm in the total map length, respectively (Figure 6). A total of 180, 173, and 170 SSR markers were distributed on genomes A, B, and D, respectively. In the prepared map, the average distance between the flanked markers for the whole genome was 9014. Chromosome 3B had the maximum map length (271.4 cm) and the highest number of markers (28 markers), and chromosome 7D had the minimum map length (147.6 cm) and the lowest number of markers (16 markers).

In the Zagros and Gonbad populations, different QTLs were identified for two years. In 2020, 12 QTLs were identified for 8 traits; in 2021, 22 QTLs were identified for 15 different traits.

In 2020, some QTLs were identified for NGS, NDF, FLL, and GW. Two QTLs were identified on chromosomes 1A and 4B for the number of germinated seeds, explaining 22.15% and 16.01% of phenotypic variation in the trait, respectively. For the number of days to spiking, a QTL was identified on chromosome 6D, explaining 30.50% of phenotypic variation. For FLL, two QTLs were identified on chromosome 1D at 70.51 and 161.52 cm, with LOD scores of 3.86 and 3.17, respectively. In 2020, qSD-2A was identified for GW on chromosome 2A, explaining 52% of phenotypic variation.

For PDL, there were three QTLs on chromosomes 1B, 4A, and 4B in 2020, and two QTLs on chromosome 1B in 2021. These QTLs were mapped at 4.85, 97.36, 193.57, 49.22, and 160.32 cm. One QTL was identified for PDL on chromosome 1B in both years. For GL, one QTL was identified on chromosome 4D with an LOD score of 2.79 in 2020, and five QTLs on chromosomes 1D (two QTLs), 3A (two QTLs), and 7D, with LOD scores of 2.2, 2.99, 3.97, 3.39, and 5.16, respectively, in 2021. For WSP, three QTLs were identified (qSW-1B in 2020, and qSW-2B and qSW-3A in 2021) on chromosomes 1B, 2B, and 3A, respectively. These QTLs explained 15.50-22.50% of phenotypic variation in WSP. A QTL was identified for TGW on chromosomes 1A and 1D in 2020 and 2021, respectively. The LOD values for these QTLs were 3.10 and 2.59, respectively.

In 2021, some QTLs were identified for the tiller number, plant height, spike length, spike weight, harvest index, and peduncle diameter. qNT-2B was identified at 37.23 cm from the top of chromosome 2B with an LOD score of 3.12 and an additive effect of 104.97 for the number of tillers.

For PH, two QTLs were identified on chromosomes 1A and 3A, explaining 16.5% and 10% of phenotypic variation in the trait, respectively. SPL on chromosome 7B at 127.75 cm and with an LOD score of 2.92 was able to explain 26.06% of phenotypic variation. In 2021, qTSW-6A was identified for total spike weight. This QTL also explained more than 31% phenotypic variation in the total WSP. The additive effect and the LOD scores were −0.195 and 2.54, respectively. For each of the PD and HI, only one QTL was identified in 2021. qPD-7B on chromosome 7B with 21.58% of phenotypic variation in a trait and qHI-7D on chromosome 7D with above 30% of phenotypic variation in a trait were recognized as the significant QTL effects (Table 3 and Table 4).

### 3.2. Gonbad Kohdasht RIL Population 

#### 3.2.1. Phenotypic Evaluations

The frequency distribution of phenotypic values of the study traits is shown in Figures S3 and S4 for the population resulting from Gonbad × Kuhdasht in 2020 and 2021, respectively. In this population, the phenotypic distribution of traits was continuous and normal. 

**Figure 7:**
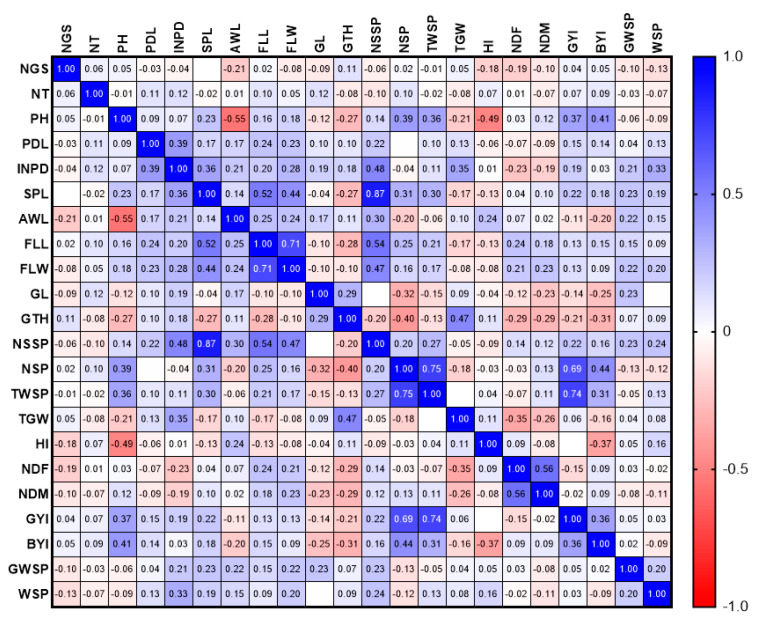
Correlation of study traits in F8 wheat lines resulting from Gonbad × Kohdasht in 2020.

The results showed that FLL had a positive and significant relationship with the traits of SPL, FLW, and NSSP in 2020 and with NSSP in 2021. In 2020, GYI had a positive and significant relationship with NSP (0.692 **) and total WSP (0.737 **). There was a positive and significant relationship between AWL, PH, and NDM (Figure 7). 

**Figure 8:**
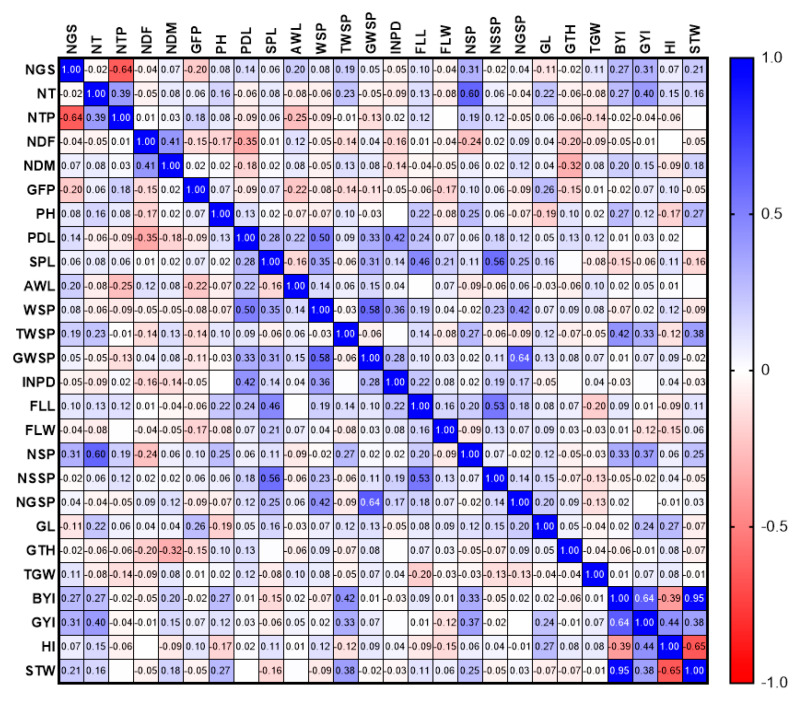
Correlation of study traits in F8 wheat lines resulting from Gonbad × Kohdasht in 2021.

In 2021, a significant and positive relationship was observed between BYI, GYI, and STW. The results showed that there is a positive and significant relationship between WSP and PDL, as well as between NSP and NT (Figure 8).

The results of stepwise regression showed that, when GYI is considered as a dependent variable and other traits as independent traits, GYI is explained by NSP and INDP (Table 5). However, NT and NGS and GL in 2020 best explained GYI (Table 6).

**Figure 9:**
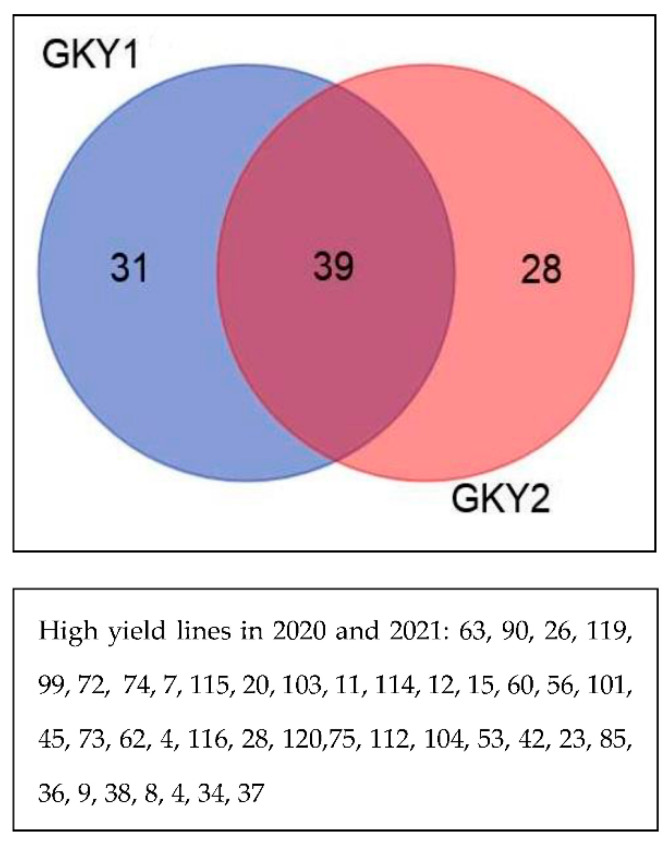
Classification of F8 wheat lines resulting from Gonbad × Kohdasht in 2020 and 2021.

The cluster analysis results divided the study lines into two groups based on study traits. In both years, lines 63, 90, 26, 119, 99, 72, 74, 7, 115, 20, 103, 11, 114, 12, 15, 60, 56, 101, and 45 had high GYI (Figure 9).

The reaction of lines caused by Gonbad × Kohdasht crosses in 2020 showed that lines 106, 103, 103, 60, and 75 were the most important in terms of PH, NDF, TGW, GYI, and NSP. Considering these traits, genotypes 26, 67, 60, and 63 were selected as the best cultivars. The traits of NDF and TGW show the most diversity for the evaluated lines.

**Figure 10:**
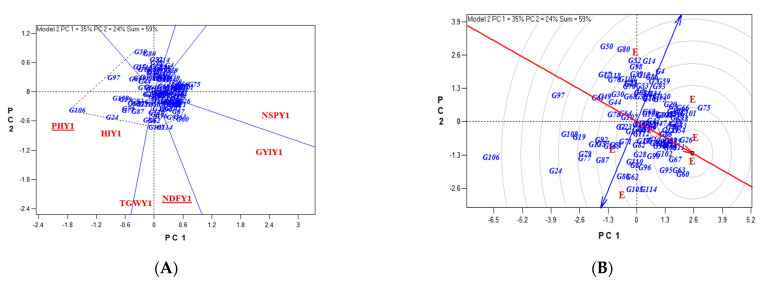

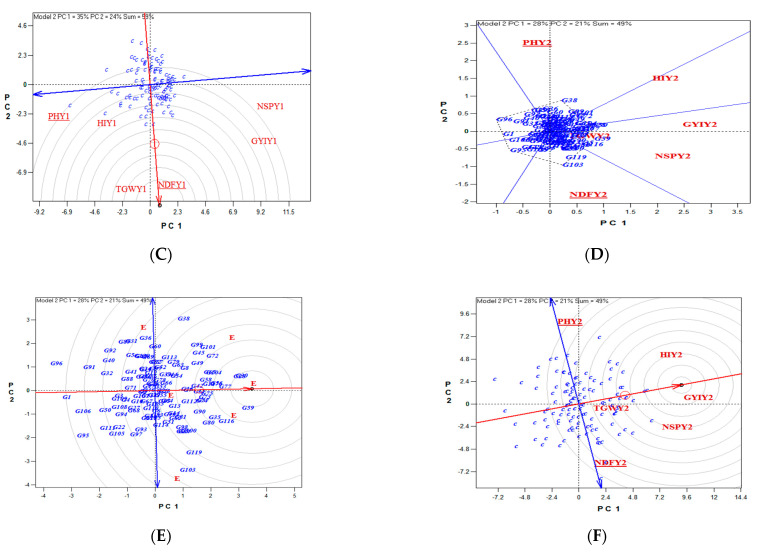
The reaction of lines caused by Gonbad × Kohdasht crosses by considering significant traits in 2020 and 2022 using Biplot. Identifying genotypes with higher values for traits (**A**,**D**), determining the best genotype considering significant traits (**B**,**E**), and identifying the most important traits affecting genetic diversity (**C**,**F**).

Moreover, Biplot analysis showed that lines 38, 103, 59, 59, and 59 were the most valuable in terms of plant height, NDF, TGW, GYI, and NSP in 2021. The genotypes 59 and 38 were selected as the best cultivars. The traits of GYI, NSP, and HI showed the most diversity for the studied lines (Figure 10).

#### 3.2.2. Genotypic Evaluations

**Figure 11:**
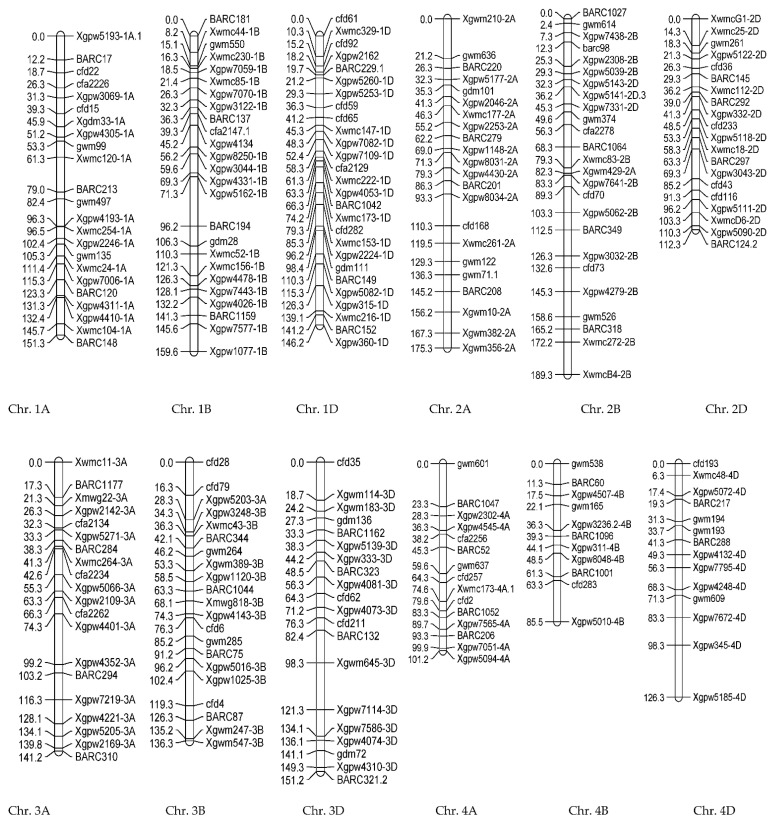

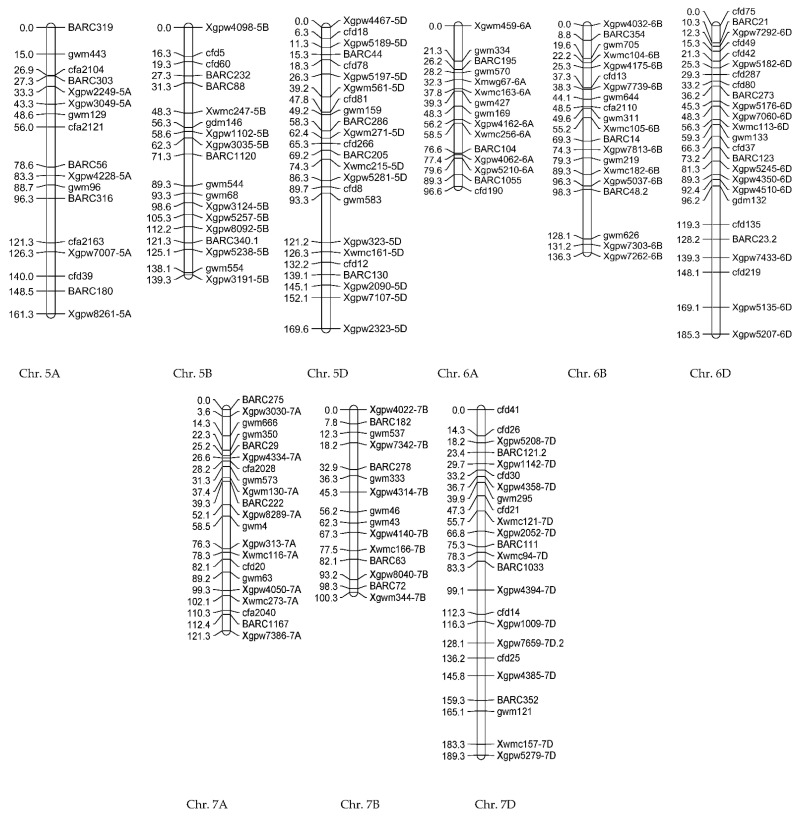
SSR linkage map developed using F8 wheat lines resulting from Gonbad × Kohdasht.

The linkage map was provided using 423 SSR markers of the genetic map. The markers were distributed on 21 wheat chromosomes. The length of this map was 2975 cm, and the average marker distances for genomes A, B, and D were 7.12, 6.96, and 5.78 cm, respectively. The share of genome A in the length of the map was 948.2 cm, and the genomes B and D were 946.9 and 890.9.9 cm of the wheat genome, respectively (Figure 11). Out of 423 SSR markers, 133, 136, and 154 SSR markers were identified on genomes A, B, and D, respectively. In the prepared map, the average distance between adjacent markers in the whole genome was 7.033. Chromosome 2B had the maximum linkage length (189.3 cm) and the highest number of markers (25 markers), and chromosome 4B had the minimum linkage length (85.5 cm) and the lowest number of markers (11 markers).

In 2020, QTLs were identified for GYI, BYI, GWSP, WSP, GW, and HI. For GYI, two QTLs were identified on chromosomes 1B and 5B. The LOD scores and their additive effects were 3.23, 3.51, −526.73, and −747.96, respectively. The QTLs controlling biological yield were identified on chromosomes 1B, 4A, and 5D, and explained 18% of phenotypic variation in the biological yield. qGWSP-2B and qGWSP-6D were identified on chromosomes 2B and 6D at 1.57 and 141.22 cm from the top of chromosomes for GWSP with 13 and 10% phenotypic variation, respectively. For spike weight, five QTLs were identified on chromosomes 1A, 5A, 7B, 2B, and 2D, explaining 5–12% of phenotypic variation in WSP.

In 2021, only one QTL was identified on chromosomes 2A, 5B, 2A, 4D, 6B, and 6B for NGS, NT, PDL, and NSP. Among these QTLs, qNT-5B with an additive effect of 0.413 and an LOD score of 4.05 could explain over 21% of phenotypic variation in a trait. For AWL, two QTLs were identified on chromosomes 4D and 6B at 53 and 17 cm, respectively. For the number of grains per spike, three QTLs were identified on chromosomes 1D, 2A, and 4B.

For GL and NSSP, several QTLs were tracked in 2020 and 2021. A QTL was identified on chromosomes 3D and 1D in 2020 and 2021, explaining 23% and 21% of phenotypic variation, respectively. For NSSP, one QTL was identified on chromosome 2A at 86 cm in 2020 and two QTLs on chromosome 6D and 4A at 57 and 97 cm, respectively, in 2021. For NSP, three QTL were identified on chromosomes 1B, 2A, and 5D in 2020, and one QTL on chromosome 2D in 2021, explaining 8, 7, 13, and 23% phenotypic variation, respectively.

For PH, two QTLs were identified on chromosomes 1A and 3A, explaining 33% and 20% of phenotypic variation in a trait, respectively. For spike length on chromosome 7B at 127.75 cm with an LOD score of 2.92, it was able to explain 52.12% of phenotypic variation. In 2021, qSTW-6A was identified for total STW. The QTLs also explained over 62% of phenotypic variation in the total spike weights. The additive effect and the LOD score were −0.195 and 2.54, respectively. In 2021, only one QTL was identified for each PDL trait and harvest index. qPD-7B on chromosome 7B explaining over 43.16% of phenotypic variation in a trait and qHI-7D on chromosome 7D explaining over 60% of phenotypic variation in a trait were recognized as the significant QTL effects.

## 4. Discussion

GYI is a complex genetic trait strongly influenced by environmental conditions. Therefore, direct selection for performance, regardless of the characteristics of its components, can have misleading results. Therefore, grain-yield-related traits should be used to increase yield [30].

Correlation analysis is an effective tool to determine the relationship between different traits in genetically diverse populations to improve the crop modification process and indicates the severity of dependence between the study traits. Breeders explain the relationship between grain yield, agronomic, and morphological traits [31,32,33].

A positive and significant correlation was reported between grain yield, the number of spikes, plant height, spike weight, spike length, the number of grains per spike, flag leaf length, and 1000-grain weight. In the present study, NSP also significantly correlated with yield [34]. In a study [35], NT, HI, and BIO had a significant positive correlation with GYI, confirming our results.

Stepwise regression is used to select the most influential independent variables in forming dependent variables, such as GYI. This method aims to create a regression equation that includes the traits explaining the most changes in total performance [36,37]. Many researchers have used stepwise regression to eliminate ineffective traits in GYI [38,39,40,41].

Adilova et al. [42] have classified wheat genotypes into four groups using cluster analysis. There was a significant difference between the groups regarding morpho-physiological traits [42]. In the present study, the lines were divided into two high-yield and low-yield groups. 

The comparison of two linkage maps showed that, among the 600 markers used, 151 markers were polymorphic for both populations and were used to prepare maps in both populations. The highest similarity belonged to chromosome 2B, which had common polymorphic markers, and the lowest similarity was related to chromosome 7B, which had five markers in common. One of the reasons for these similarities can be pointed to a common parent of the two populations.

Marza et al. [43] have examined a wheat RIL population resulting from Clarkxning and 7840 crosses in several environments. In the present study, QTLs for grain yield and their components were identified on chromosomes A1, B1, B2, B3, A4, B4, A5, B5, B6, A7, and D7 of the bread wheat genome. Sourdille et al. [44], in the study on genetic loci associated with major agronomic traits of wheat, have identified four QTLs controlling plant height. Marza et al. [43] has identified five significant QTL effects of 16.7, 16.9, 12.3, 14.9, and 12.1 for plant height in wheat on chromosomes BS2, BL2, D2, DL2, and A6, respectively.

Sourdille et al. [44] have identified the genetic loci of wheat height. Four QTLs have been reported for plant height. Keller et al. [45] have examined QTLs controlling lodging resistance and identified 11 QTLs for plant height. A QTL with R^2^ above 20% was identified in this study on chromosome A3. Identifying this QTL on chromosome 3A could increase the reliability of our result.

Borner et al. [46] have reported a QTL on chromosome 6A for peduncle length. An obvious point about the identified QTLs is identifying QTL controlling the peduncle length in both years on chromosome 1B, indicating the stability of QTL and confirming the results. Borner et al. [46] have identified two QTLs for spike length on chromosomes D2 and B6, whereas, in the present study, only one QTL was identified for spike length on chromosome 7B in Gonad × Zagros in 2021.

In the present study, three QTLs were identified for the number of grains per spike in the population resulting from Gonbad × Kuhdasht in 2021, two of which were on chromosomes 2A and 4B, under different environmental conditions. As QTLs did not appear in the same region on the chromosome under different environmental conditions, it can be concluded that the interaction of QTLs in the environment regarding the number of grains per spike is significant. Sishen et al. [47] have also identified several gene loci for the number of spikelets per spike. Huang et al. [48] have identified several QTLs on chromosomes 1 BL, 2AL, 2DL, 3BS, 4DS, 5DL, 6DL, and 7AS for spike. Kumar et al. [49] mapped three QTLs for tiller number, two QTLs for biological yield, and three QTLs for harvest index.

## 5. Conclusions

QTLs are significant in growth and phenotype development, but are not the only factor. Plant phenotypes are prejudiced by several environmental factors, including temperature and water availability. Stable and unstable QTLs in different environments or populations strongly influenced by environmental conditions have emerged as a common feature of quantitative traits in previous QTL studies [50]. Moreover, genetic factors, such as epigenetics, affect phenotypes. Epigenetics acts as a connection between environment and gene expression. Epigenetics refers to reversible inherited changes that occur without altering the DNA sequence. In the present study, QTL mapping was employed to dissect the genetic bases of morpho-phenological traits under two years using two populations. In our study, many significant QTLs were identified for the traits in each population in 2020 and 2021. These QTLs differed in size and phenotypic variation ratio (Table 7 and Table 8).

We found many QTLs with minor and significant genetic effects in only one year. Table 3, Table 4, Table 7 and Table 8 report the flanking marker intervals of all QTLs identified in different years and those resulting from different rain, temperatures, and other meteorological factors. In two years, only one QTL (qPDL-1B) was identified for PDL during both years on the same chromosome. It should be noted that qPDL-1B identified under deferent meteorological factors were in the same genetic region (Table 3 and Table 4).

Apparently, the environmental effect on QTL arises from differential gene expressions in different environments and may occur in any of the following three situations: (1) a QTL is expressed in one environment, but not in another; (2) a QTL is expressed strongly in one environment, but weakly in another, as indicated by the variation in its effects across environments; and (3) a QTL is expressed very differently with opposite effects in different environments [51,52]. As previously reported, one and three cases were also observed in our study.

However, in this study, we identified significant QTLs that can be used in marker-assisted selection wheat breeding programs thanks to the high percentage of explanations of changes in each trait. It is expected that, after the result validation in different places and populations, the QTLs identified in this study can be used in marker-assisted selection.

## Figures and Tables

**Table 1:** Results of stepwise regression of grain yield per hectare as a dependent variable and other traits as the independent variables in the F8 population resulting from Gonbad × Zagros in 2020.

Traits	Intercept	Coefficients		Std. Error	F	R^2^
		b1	b2			
NSP	1854.461	43.909 **		1044.419	160.974 **	0.577
TGW	−1397.54	44.152 **	83.025 **	1018.535	88.166 **	0.594

** significant at the 0.01 probability levels.

**Table 2:** Results of stepwise regression of grain yield per hectare as a dependent variable and other traits as the independent variables in the F8 population resulting from Gonbad × Zagros in 2021.

Traits	Intercept	Coefficients					Std. Error	F	R^2^
		b1	b2	b3	b4	b5			
NT	3682.314	6.156 **					1005.028	18.641 **	0.276
NGS	1678.096	6.016 **	9.014 **				850.764	23.197 **	0.491
STW	946.991	5.345 **	8.019 **	0.108 *			806.549	19.343 **	0.552
FLW	2121.728	5.144 **	7.257 **	0.121 **	−46,073.598 *		759.584	18.104 **	0.612
PD	−1864.79	5.400 **	7.249 **	0.124 **	−56,296.787 **	1591.834 **	680.978	20.466 **	0.695

* and ** significant at the 0.05 and 0.01 probability levels, respectively.

**Table 3:** QTLs identified using the F8 population derived from Gonbad × Zagrosin 2020.

Trait		Chr	Position (cM)	Additive Effect	LOD	Left_Marker	Right_Marker	R^2^ (%)
NGS	qNGS-1A	1A	102.29	−17.440	3.42	Xwmc744-1A	cfa2219	22.15
	qNGS-4B	4B	109.25	14.8282	3.15	gwm113	cfd283	16.01
PDL	qPDL-1B	1B	4.85	1.764	3.11	gwm374.1	gwm374.1	13.775
	qPDL-4A	4A	97.36	1.5644	2.73	Xgpw7543-4A	Xgpw7543-4A	10.835
	qPDL-4B	4B	193.57	−1.663	2.76	gwm495	gwm495	12.245
FLL	qFLL-1Da	1D	70.51	1.2198	3.86	BARC169	Xwmc147-1D	17.625
	qFLL-1Db	1D	161.52	−1.2523	3.17	gwm232	Xgpw4311-1D	18.575
GL	qGL-4D	4D	196.25	0.6967	2.79	BARC48	BARC288	27.985
GW	qGW-2A	2A	49.62	0.1408	3.31	BARC220	BARC220	26.035
NDF	qNDF-6D	6D	0	2.52	2.97	Xgpw7292-6D	Xgpw7292-6D	30.635
TGW	qTGW-1D	1A	177.28	2.6711	3.10	Xgpw7258-1A	BARC287	28.495
WSP	qWSP-2B	1B	74.358	0.3048	2.59	gdm28	BARC80	22.91

**Table 4:** QTLs identified using the F8 population derived from Gonbad × Zagros in 2021.

Trait		Chr	Position (cM)	Additive Effect	LOD	Left_Marker	Right_Marker	R^2^ (%)
NT	qNT-2B	2B	37.23	104.097	3.12	BARC00	gwm429	27.645
PH	qPH-1A	1A	82.36	−6.064	2.99	Xwmc93-1A	Xwmc93-1A	16.635
	qPH-3A	3A	53.56	−4.763	2.93	BARC57	cfa2262	10.265
PDL	qPDL-1Ba	1B	49.22	2.292	2.59	Xwmc85-1B	Xwmc85-1B	16.94
	qPDL-1Bb	1B	160.32	−2.180	2.68	Xgpw3190-1B	BARC302	15.325
SPL	qSPL-7B	7B	127.75	0.777	2.92	Xwmc335-7B	gwm302	26.06
WSP	qWSP-2B	2B	73.25	0.224	3.45	gwm630	gwm630	19.585
	qWSP-3A	3A	15.65	−0.202	3.94	Xgpw4221-3A	Xgpw2266-3A	15.925
TWSP	qTWSP-6A	6A	12.36	200.093	4.04	BARC171	BARC171	31.365
GWSP	qGWSP-7A	7A	121.47	−0.195	2.54	cfa2257	cfa2257	21.36
GW	qGW-7B	7B	209.45	0.216	2.12	gwm611	Xwmc792-7B	21.58
GL	qGL-1Da	1D	67.69	−2.630	2.99	BARC169	Xwmc147-1D	6.875
	qGL-3Aa	3A	76.32	−3.134	3.39	Xwmc640-3A	Xgpw7213-3A	9.765
	qGL-1Db	1D	37.23	−1.828	2.77	Xwmc489-1D	Xwmc489-1D	3.32
	qGL-3Ab	3A	44.28	2.393	3.97	BARC57	BARC57	5.69
	qGL-7D	7D	78.32	3.345	5.16	Xgpw4385-7D	gdm145	11.12
TGW	qTGW-1D	1D	70.51	2.041	2.59	BARC169	Xwmc147-1D	22.54
HI	qHI-7D	7D	35.22	6.22	3.18	cfd41	Xgpw2160-7D	30.235
NFSP	qNFSP-5D	5D	44.24	−0.28	2.07	BARC143	Xgpw7238-5D	18.765
NDF	qNDF-4A	4A	18.09	−2.603	3.37	Xgpw4545-4A	BARC106	26.095
NDM	qNDM-6B	6B	174.36	−1.452	2.65	gwm626	gwm626	25.99
GFP	qGFP-5D	5D	138.29	−1.966	2.89	Xwmc264-5D	cfd7	23.185

**Table 5:** Results of stepwise regression of grain yield per hectare as a dependent variable and other traits as the independent variables in the F8 population resulting from Gonbad × Kohdasht cross in 2020.

Traits	Intercept	Coefficients		Std. Error	F	R^2^ (%)
		b1	b2			
NSP	2671.308	37.511 **		1142.479	108.576 **	0.679
PD	−557.581	37.939 **	1201.858 **	1093.953	65.065 **	0.726

** significant at 0.01 probability levels.

**Table 6:** Results of stepwise regression of grain yield per hectare as a dependent variable and other traits as the independent variables in the F8 population resulting from Gonbad × Kohdasht cross in 2021.

Traits Entered in Mode	Intercept	Coefficients			Std. Error	F	R^2^ (%)
		b1	b2	b3			
NT	4584.416	4.059 **			1022.691	21.971 **	0.157
NGS	3179.422	4.136 **	5.922 **		963.953	20.274 **	0.257
GL	2183.118	3.682 **	6.310 **	78.775 *	943.137	16.193 **	0.295

* and ** significant at the 0.05 and 0.01 probability levels, respectively.

**Table 7:** QTLs identified using the F8 population derived from Gonbad × Kohdasht in 2020.

Trait	QTL	Chr	Position (cM)	Additive Effect	LOD	Left_Marker	Right_Marker	R^2^ (%)
NGS	qNGS-6B	6B	33.26	−8.0386	2.76	Xgpw4175-6B	cfd13	18.91
FLL	qFLL-1Db	3A	40.26	0.6082	2.89	BARC284	Xwmc264-3A	18.05
GL	qGL-3D	3D	110.25	0.2817	2.70	Xgwm645-3D	Xgpw7114-3D	23.92
GW	qGW-2A	2A	144.17	−0.0598	2.56	gwm71.1	BARC208	9.90
NSSP	qNSSP-2A	2A	86.25	0.3751	2.88	BARC201	BARC201	11.07
NSP	qNSP-1B	1B	45.23	8.8226	3.07	Xgpw4134	Xgpw4134	8.72
	qNSP-2A	2A	119.54	−8.4177	2.68	Xwmc261-2A	Xwmc261-2A	7.94
	qNSP-5D	5D	1.80	−10.8852	2.77	Xgpw4467-5D	cfd18	13.28
HI	qHI-1A	1A	108.75	−3.8254	3.15	gwm135	Xwmc24-1A	21.35
GYI	qGYI-1B	1B	0	−526.736	3.23	BARC181	BARC181	9.71
	qGYI-5B	5B	102.39	−747.969	3.51	Xgpw3124-5B	Xgpw5257-5B	19.5
BYI	qBYI-1B	1B	32.26	1579.43	3.03	Xgpw3122-1B	Xgpw3122-1B	8.23
	qBYI-4A	4A	74.59	−1627.6	3.04	Xwmc173-4A.1	Xwmc173-4A.1	8.74
	qBYI-5D	5D	130.18	−2353.14	4.05	Xwmc161-5D	cfd12	18.28
GWSP	qGWSP-2B	2B	1.57	0.0966	5.71	BARC1027	gwm614	13.35
	qGWSP-6D	6D	141.22	−0.0853	5.09	Xgpw7433-6D	cfd219	10.41
WSP	qWSP-2B	1A	108.75	−0.3722	3.07	gwm135	Xwmc24-1A	12.81
	qWSP-2B	5A	37.26	0.331	2.71	Xgpw2249-5A	Xgpw3049-5A	10.13
	qWSP-2B	7B	39.86	0.3526	2.51	gwm333	Xgpw4314-7B	11.49
	qWSP-2B	2B	83.26	−0.2552	3.06	Xgpw7641-2B	Xgpw7641-2B	6.02
	qWSP-2B	2D	103.26	0.2436	2.59	XwmcD6-2D	XwmcD6-2D	5.48

**Table 8:** QTLs identified using the F8 population derived from Gonbad × Kohdasht in 2021.

Trait		Chr	Position (cM)	Additive Effect	LOD	Left_Marker	Right_Marker	R^2^ (%)
NSG	qNSG-2A	2A	62.23	22.2059	3.23	BARC279	BARC279	13.67
NT	qNT-5B	5B	64.30	0.413	4.05	Xgpw3035-5B	BARC1120	21.16
PL	qPL-2A	2A	28.82	1.0326	2.47	BARC220	Xgpw5177-2A	15.83
AWL	qOL-4D	4D	53.65	0.3434	2.44	Xgpw4132-4D	Xgpw7795-4D	12.79
	qOL-6B	6B	17.61	−0.3967	2.91	BARC354	gwm705	17.06
STW	qSTW-6B	6B	42.16	−94.138	2.37	Xgpw7739-6B	gwm644	18.35
NTS	qNTS-2D	2D	43.05	77.9566	4.13	Xgpw332-2D	cfd233	23.87
NSP	qNSP-6D	6D	57.25	0.6214	2.71	Xwmc113-6D	gwm133	19.53
	qNSP-4A	4A	97.96	0.4065	2.44	BARC206	Xgpw7051-4A	8.36
NGSP	qNGSP-1D	1D	49.9	3.145	3.52	Xgpw7082-1D	Xgpw7109-1D	15.25
	qNGSP-2A	2A	173.50	−2.5984	2.69	Xgwm382-2A	Xgwm356-2A	10.41
	qNGSP-4B	4B	8.43	2.9969	3.12	gwm538	BARC60	13.85
GL	qGL-1D	1D	49.9	1.3773	3.89	Xgpw7082-1D	Xgpw7109-1D	21.63

## Data Availability

The data presented in this study are available upon request from the corresponding authors.

## Supplementary Materials

The following supporting information can be downloaded at https://www.mdpi.com/article/10.3390/biotech11030032/s1, Figure S1: Frequency distribution of observed values of study traits in F8 wheat lines resulting from Gonbad × Zagros in 2020; Figure S2: Frequency distribution of observed values of study traits in F8 wheat lines resulting from Gonbad × Zagros in 2021; Figure S3: Frequency distribution of observed values of study traits in F8 wheat lines resulting from Gonbad × Kohdasht in 2020; Figure S4: Frequency distribution of observed values of study traits in F8 wheat lines resulting from Gonbad × Kohdasht in 2021; Table S1: Meteorological statistics from Gonbad Kavous Agricultural Research Station in 2020; Table S2: Meteorological statistics from Gonbad Kavous Agricultural Research Station in 2021.
