# Supplementary material for: SSR Linkage Maps and Identification of QTL Controlling Morpho-Phenological Traits in Two Iranian Wheat RIL Populations
Source: BioTech (Basel). 2022 Aug 8;11(3):32. doi: 10.3390/biotech11030032 (PMC9397039; doi:10.3390/biotech11030032)
Supplement: Supplementary file 1 [file biotech-11-00032-s001.zip › Supplementary Figures.pdf]

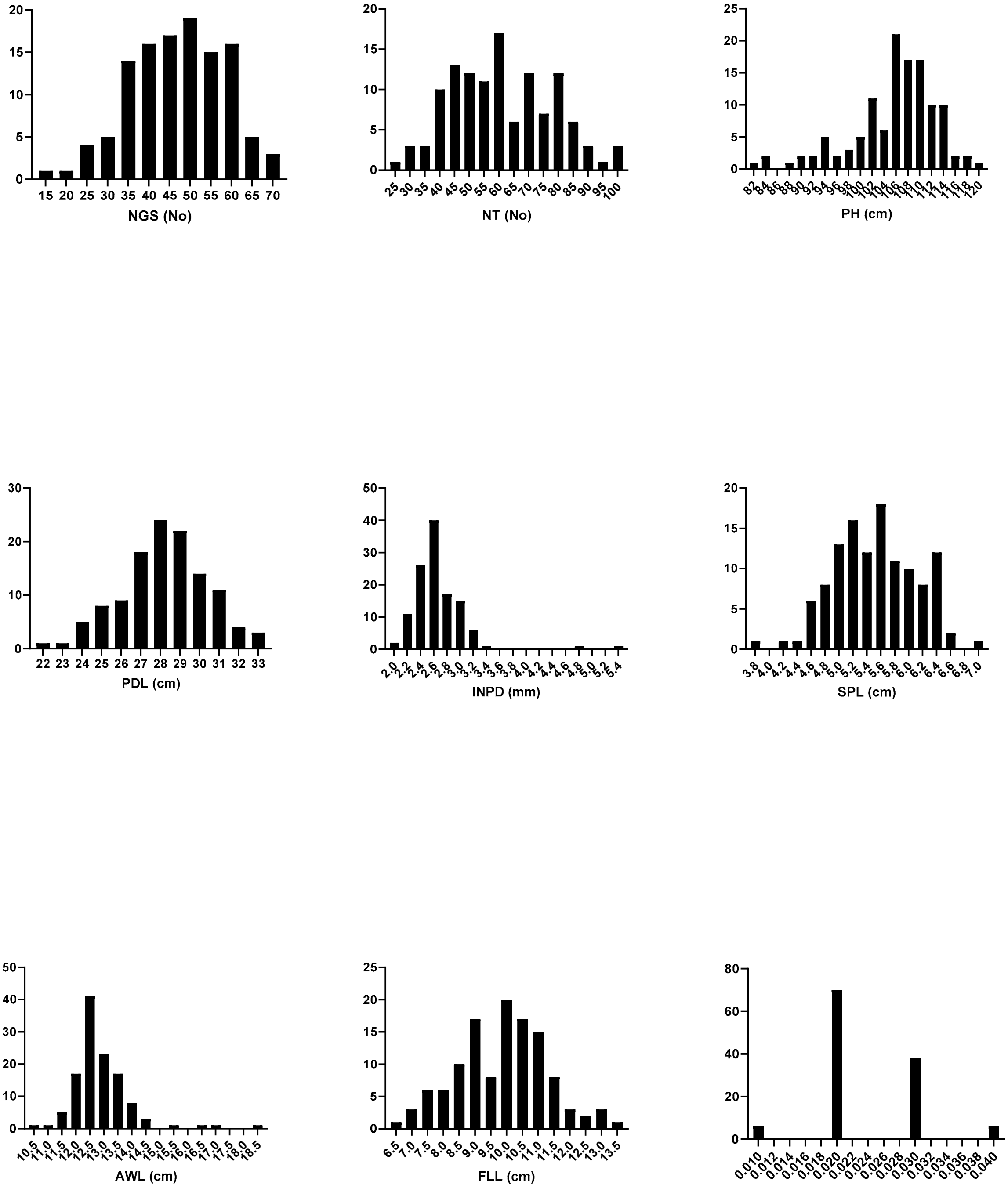

Figure S1. Frequency distribution of observed values of studied traits in F8 lines of wheat obtained from Gonbad × Zagros in 2020

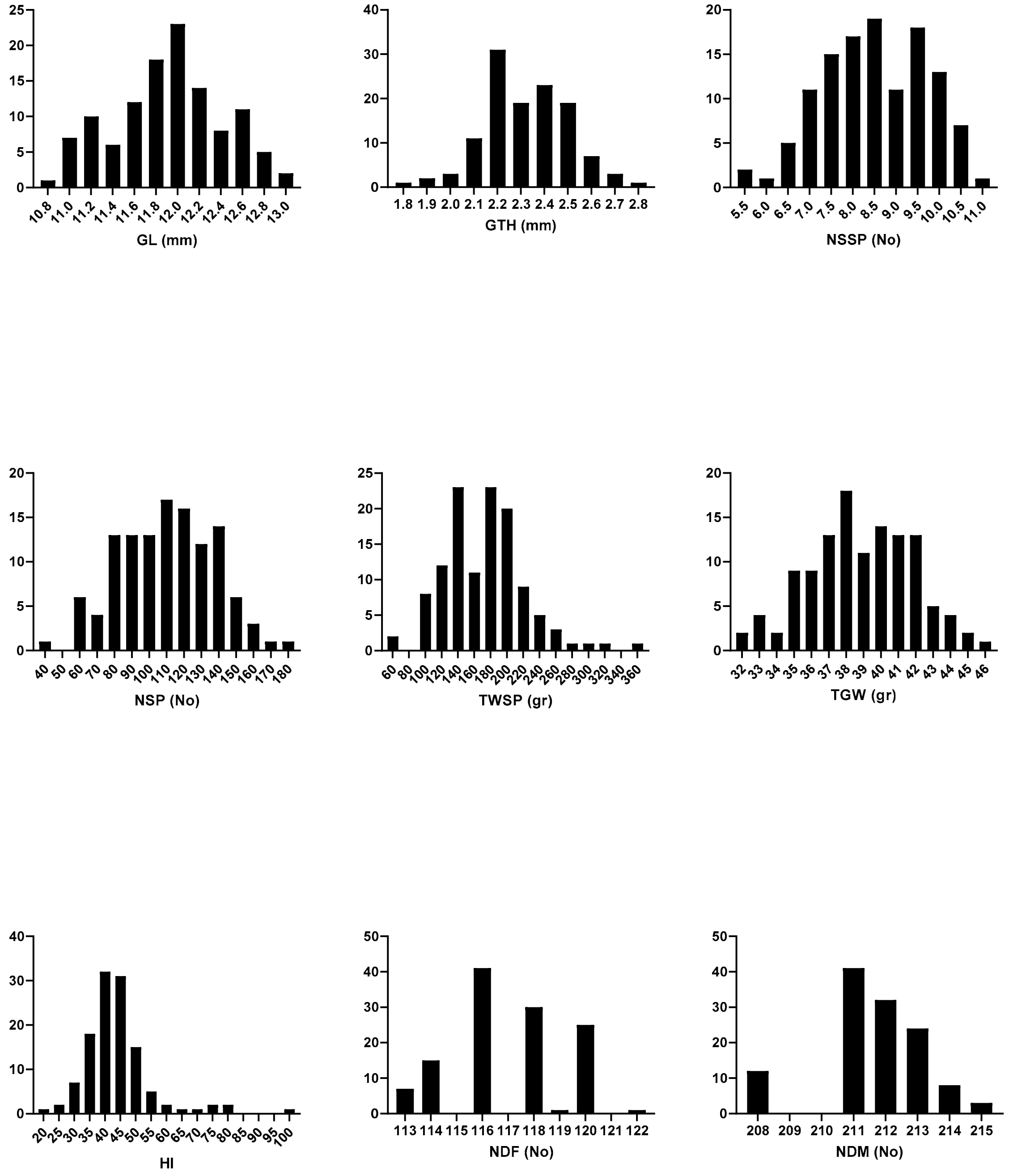

Continue Figure S1.

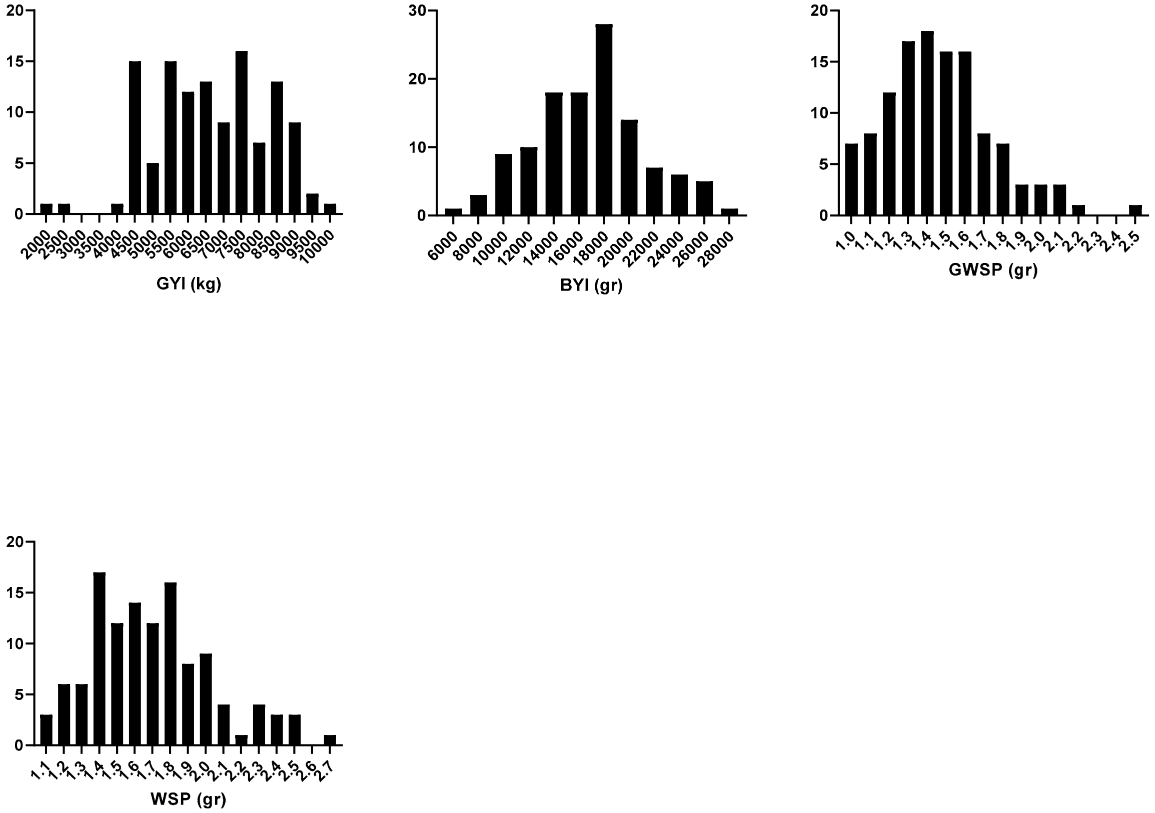

Continue Figure S1.

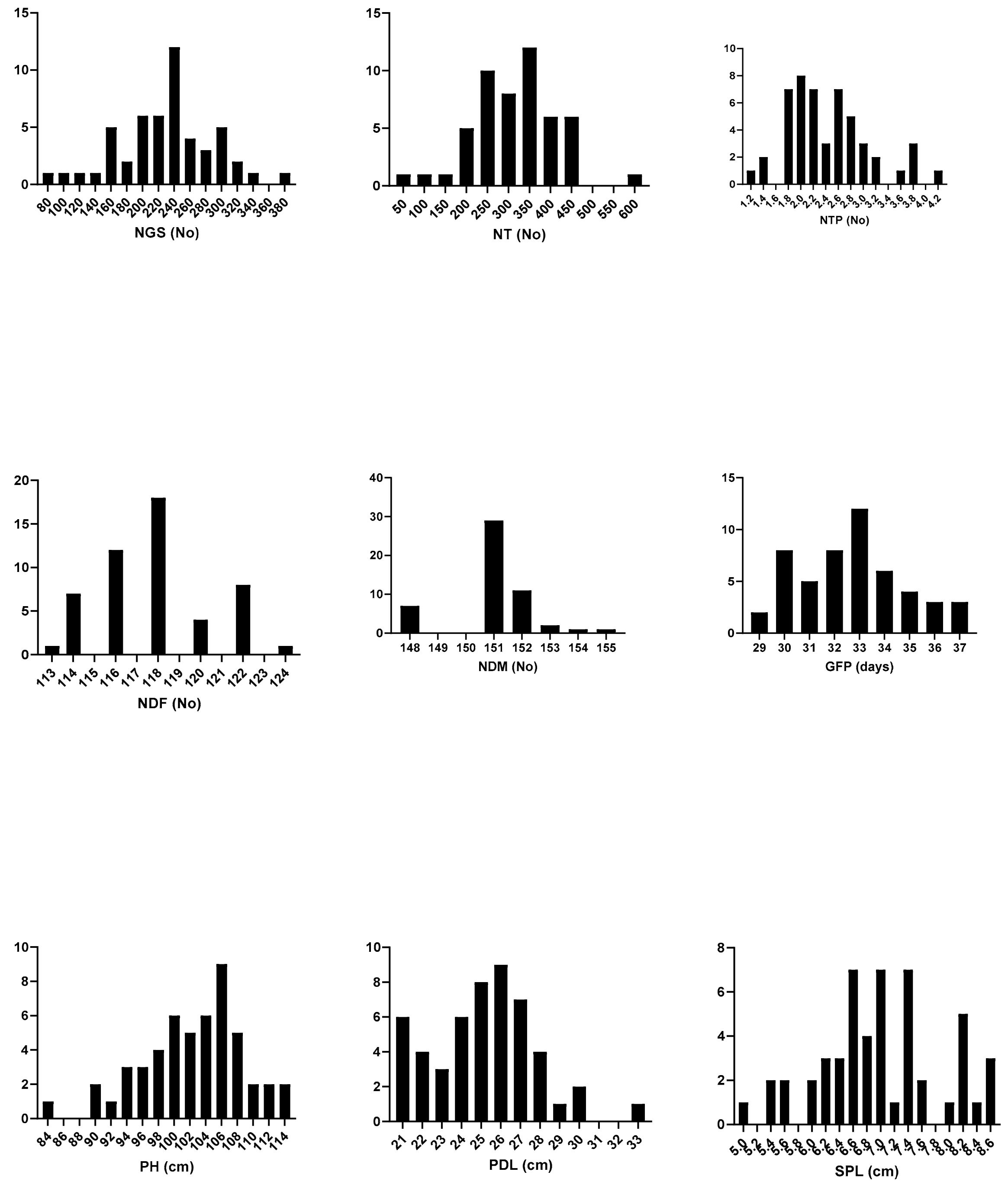

Figure S2. Frequency distribution of observed values of studied traits in F8 lines of wheat obtained from Gonbad × Zagros in 2021

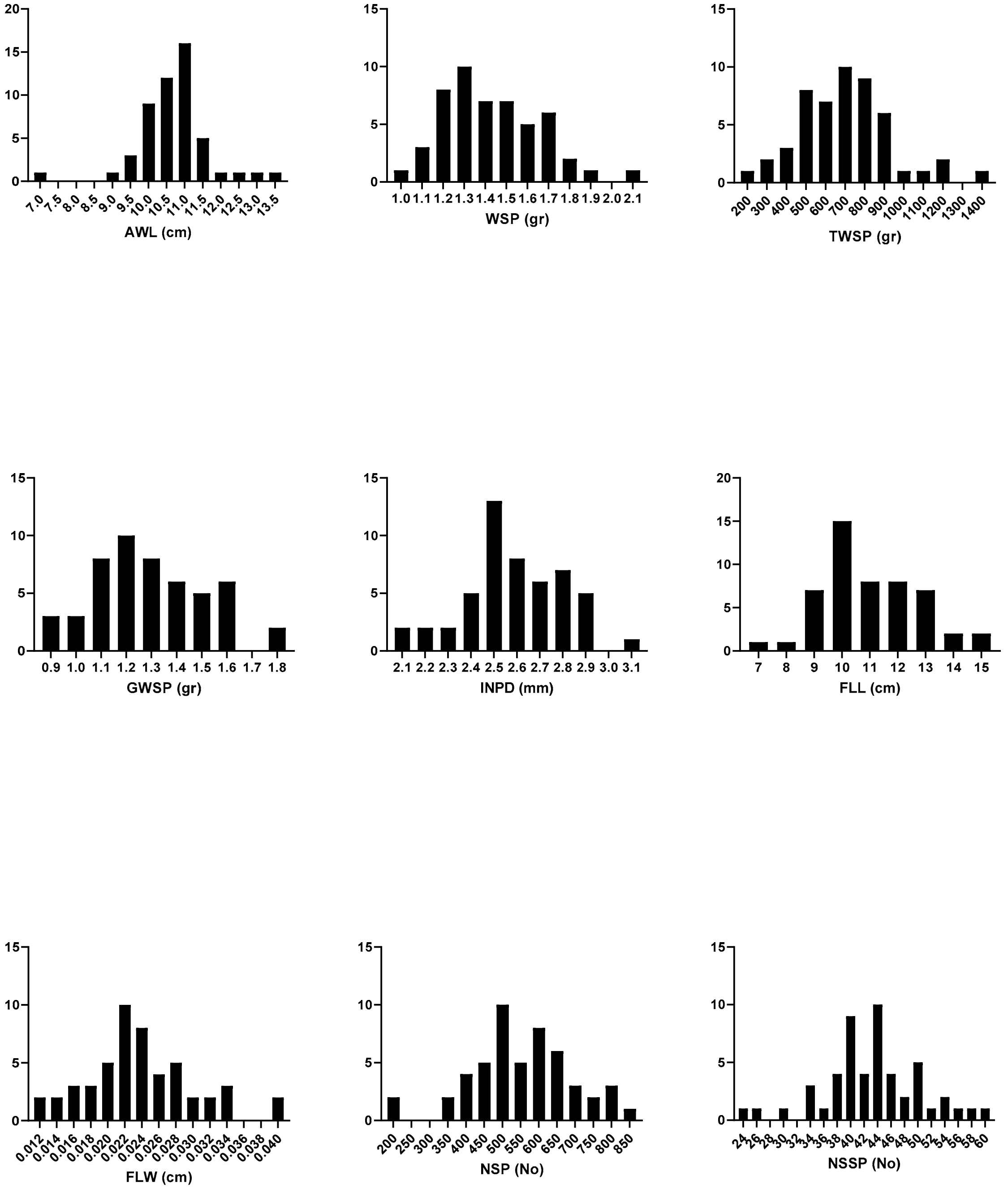

Continue Figure S2

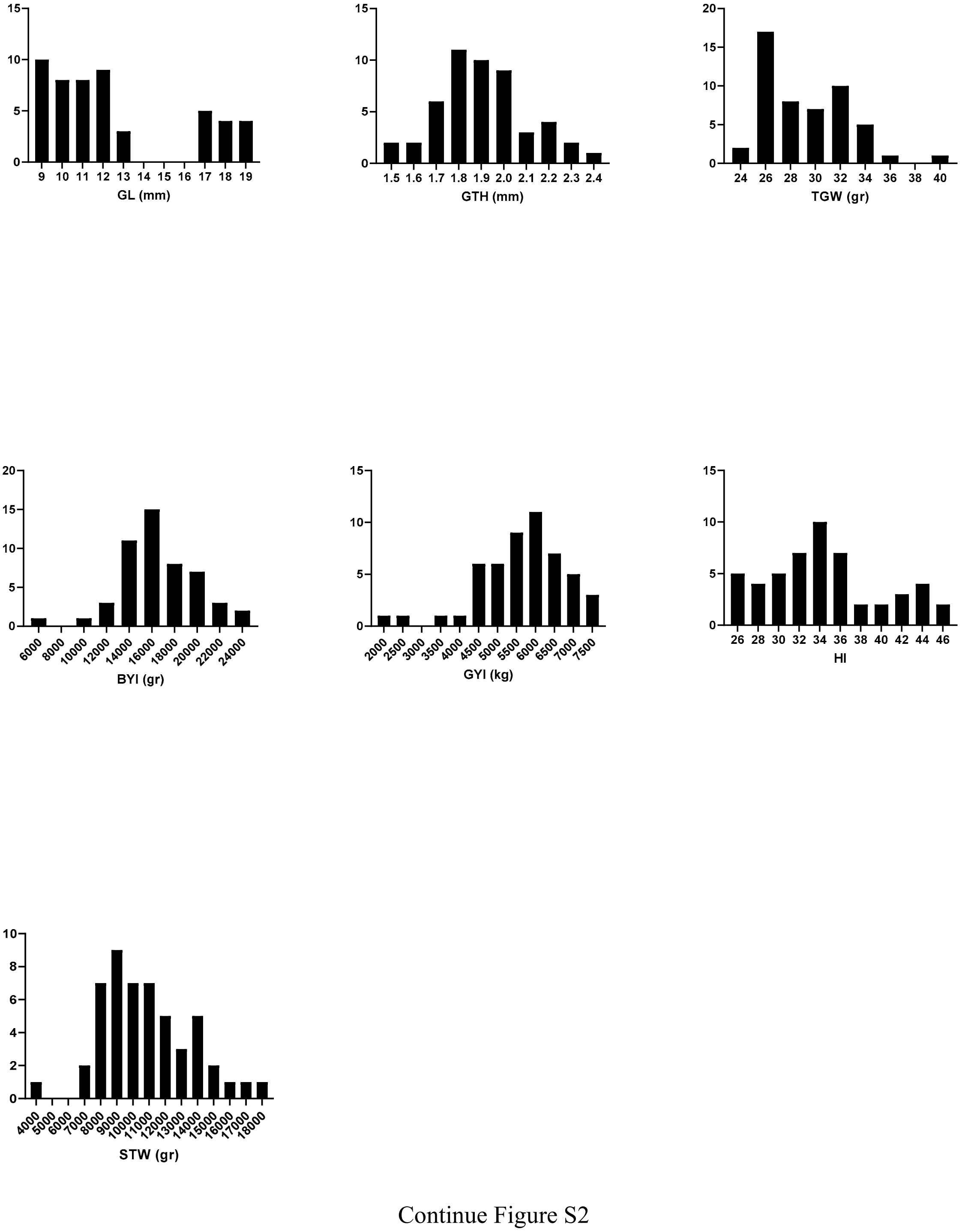

Continue Figure S2

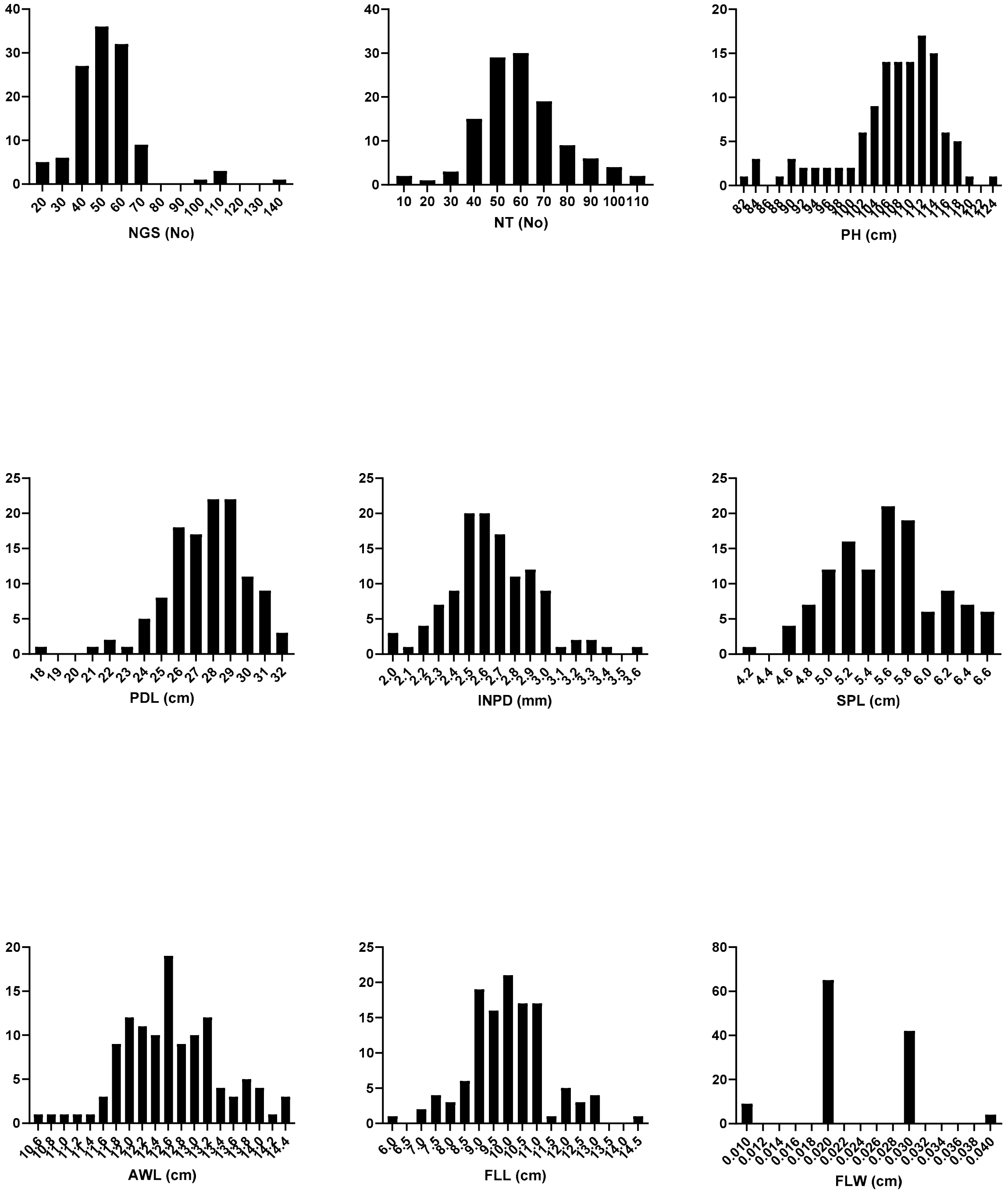

Figure S3. Frequency distribution of observed values of studied traits in F8 lines of wheat obtained from Gonbad × Kohdasht in 2020

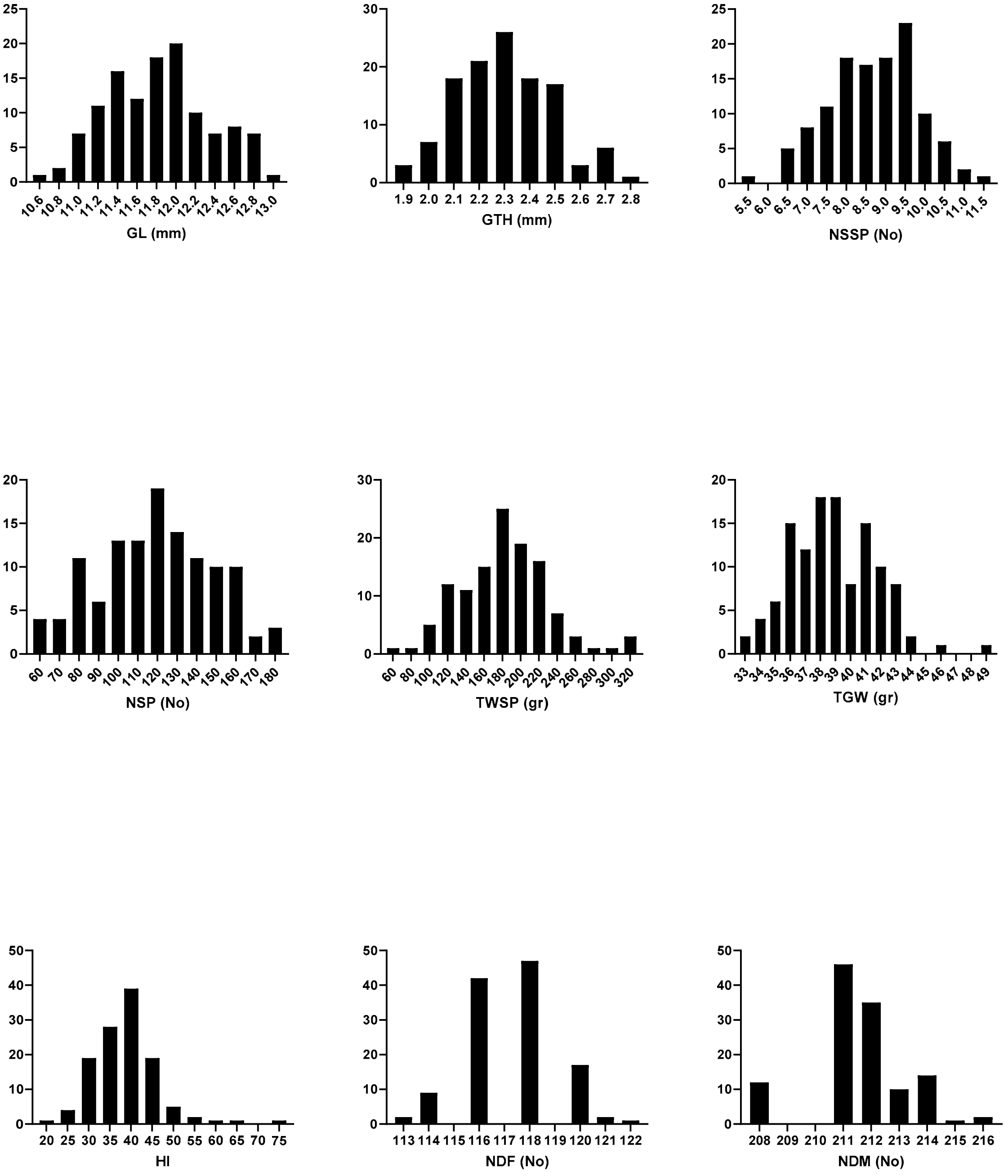

Continue Figure S3.

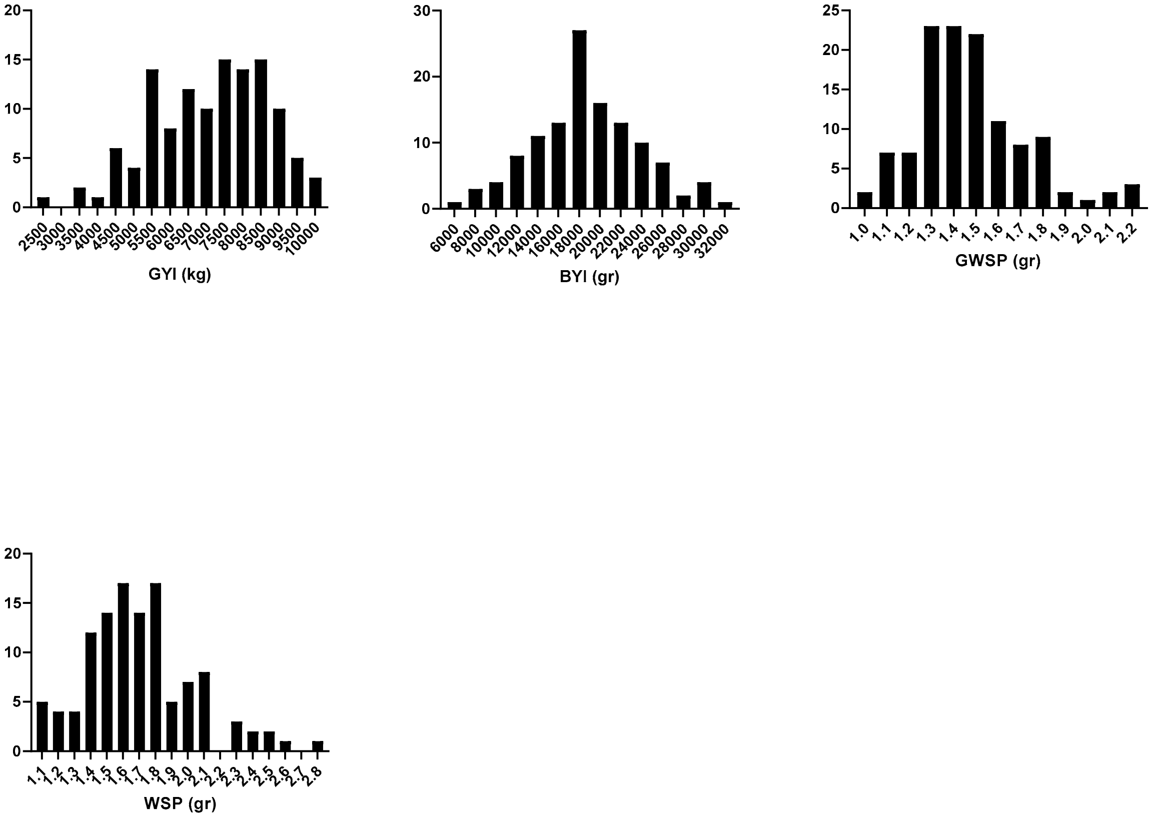

Continue Figure S3.

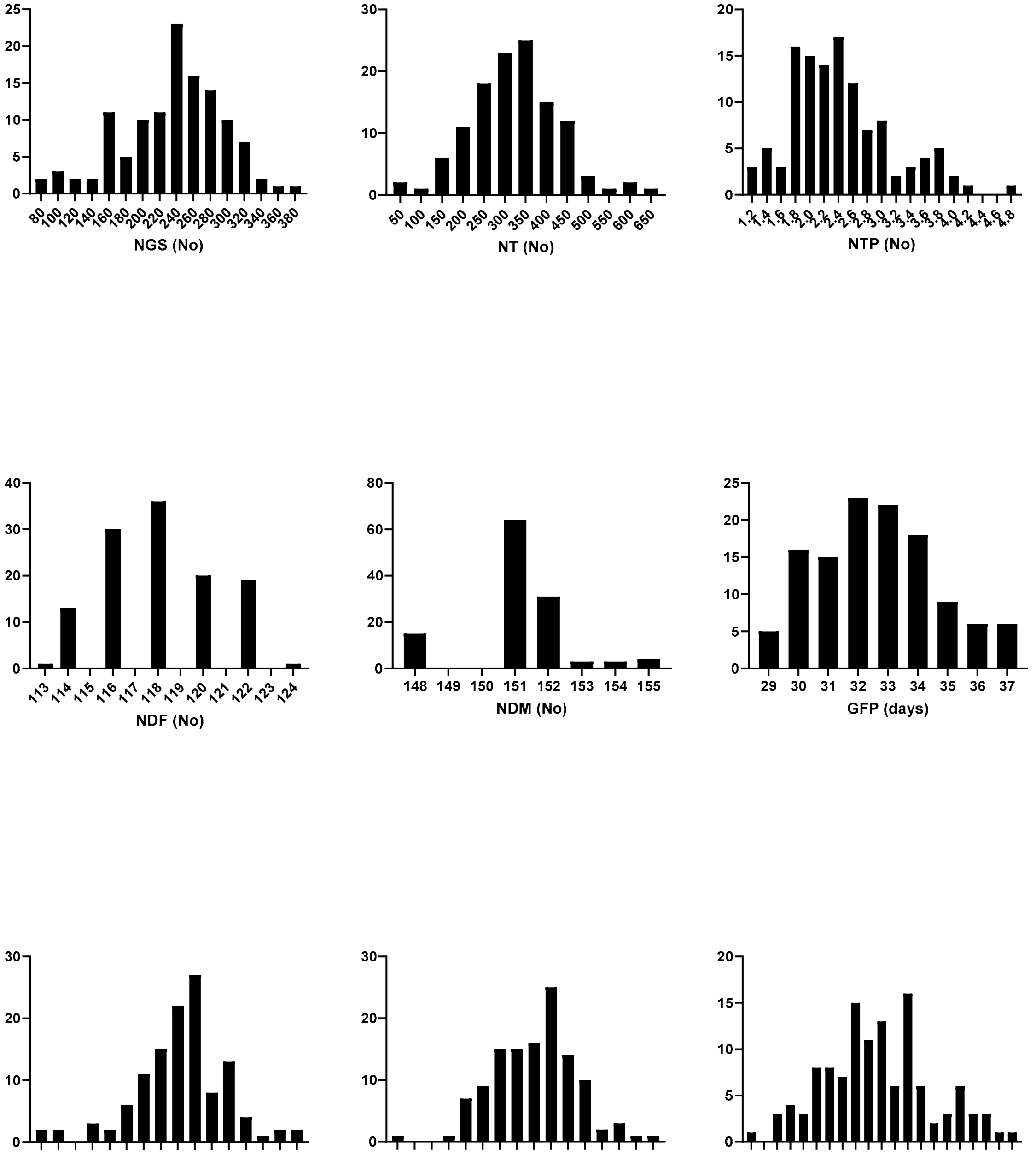

Figure S4. Frequency distribution of observed values of studied traits in F8 lines of wheat obtained from Gonbad × Kohdasht in 2021

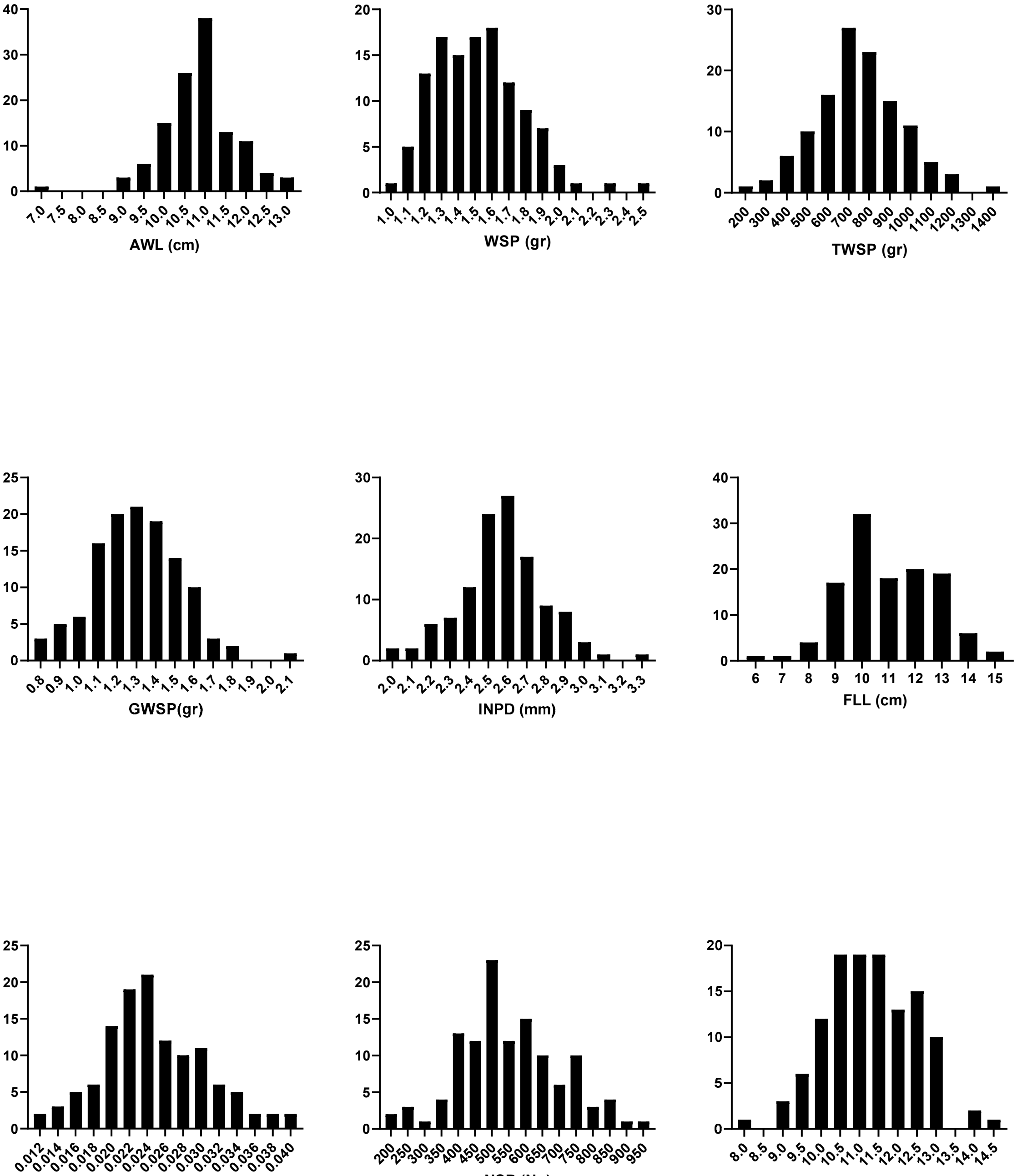

Continue Figure S4.

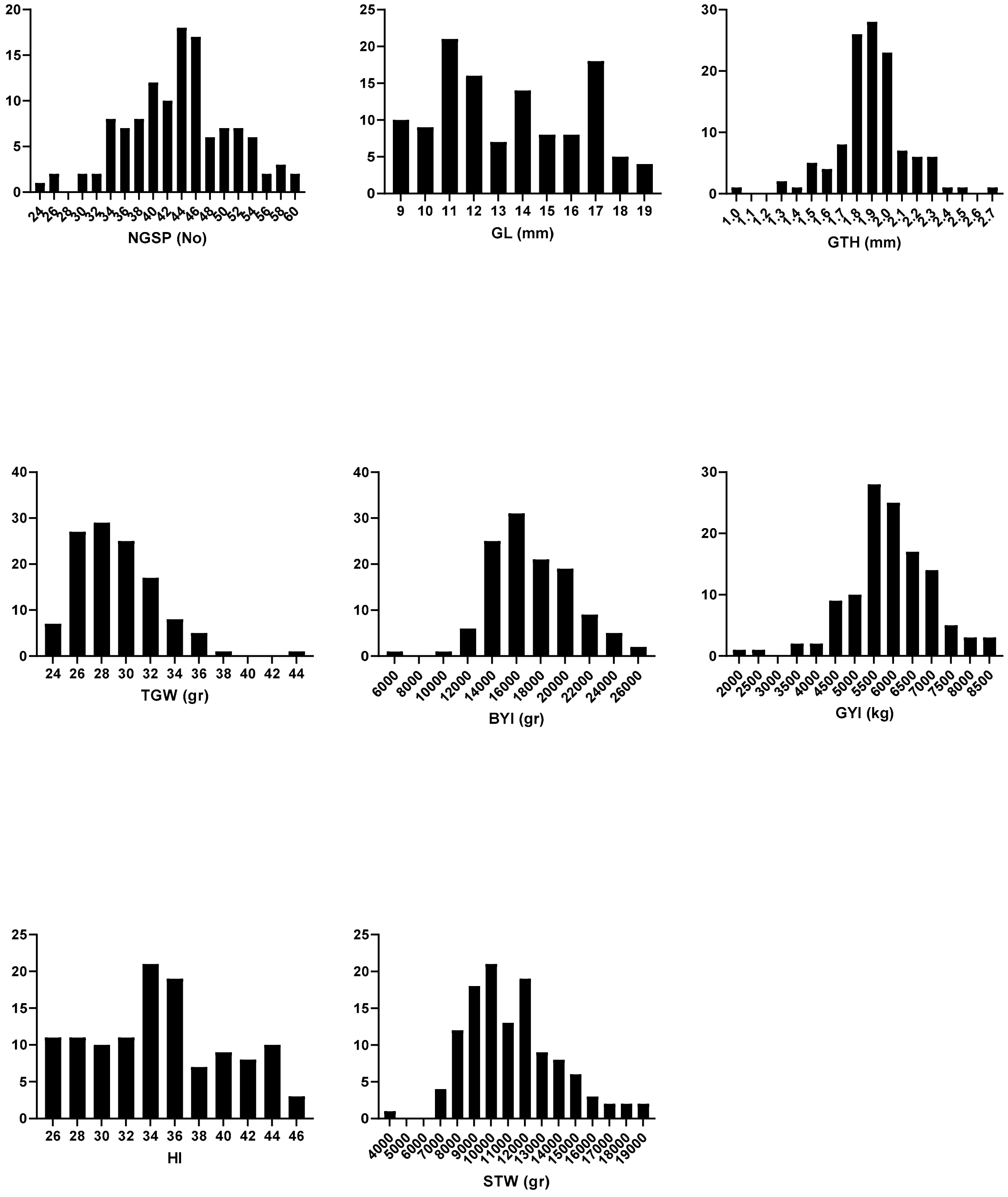

Continue Figure S4.
