# Supplementary material for: SSR Linkage Maps and Identification of QTL Controlling Morpho-Phenological Traits in Two Iranian Wheat RIL Populations
Source: BioTech (Basel). 2022 Aug 8;11(3):32. doi: 10.3390/biotech11030032 (PMC9397039; doi:10.3390/biotech11030032)
Supplement: Supplementary file 1 [file biotech-11-00032-s001.zip › Supplementary Tables.pdf]

Table S1. Meteorological statistics Gonbad Kavous Agricultural Research Station in 2020

Month	rain (mm)	Min absolute temperature	Max absolute tempe	Average temperature	Number of days below zero	Relative humidity %	evaporation (mm)	Average min temperature	Average max temperature
Oct	22.9	7.7	36	22.3	-	61	113.2	14.4	30.3
Nov	56.4	1.7	30.9	15.1	-	72	48.3	8.6	21.6
Dec	11.9	-0.5	16.8	11.8	1	74	38	5.4	18.1
Jan	16.4	-0.5	28.8	10.4	1	66	43	3.8	17.1
Feb	68.4	-2.1	31.7	9.9	6	64	51.9	2.8	17
Mar	65.9	0.4	26.9	12.4	-	77	46.1	5.8	19
Apr	93.2	0	30.3	13.7	1	81	51.4	8.2	19.3
May	40.6	8.4	36.1	19.6	0	73	99.5	12.9	26.3
June	2.4	12.8	46.9	27.6	0	48	225.4	18.7	36.4
Total	398	-2.1	46.9	17.2	9	66.5	966	10.28	23.77

Table S2. Meteorological statistics Gonbad Kavous Agricultural Research Station in 2021

**Month**	**rain (mm)**	**Min absolute temperature**	**Max absolute tempe**	**Average temperature**	**Number of days below zero**	**Relative % humidity**	**evaporation (mm)**	**Average min temperature**	**Average max temperature**
Oct	30.4	6.9	32.4	20.3	0	57	112.12	13	27.6
Nov	16.5	1.5	37.2	16.5	0	66	70.1	10.1	23
Dec	31.9	-4	20	8.9	4	78	24.8	4.2	13.6
Jan	31.8	-3.1	29.6	8.3	9	72	40.2	1.9	14.7
Feb	24.5	-3.9	30.1	10.2	6	71	47.1	2.9	17.6
Mar	62.2	-5.7	35.9	9.1	11	73	43.6	2.7	15.5
Apr	16.6	2.6	34.3	16.6	0	67	99.1	9.5	23.6
May	20	12.2	43.8	22.3	0	62	149	14.9	29.8
June	12.2	14.8	46.5	28.2	0	50	225.9	20.1	36.3
Total	246.1	-5.7	46.5	15.6	30	66.22	716.8	8.81	22.41
